# It is possible to make patients use braces the hours prescribed: first results from the thermobrace clinical everyday usage

**DOI:** 10.1186/1748-7161-7-S1-O29

**Published:** 2012-01-27

**Authors:** S Donzelli, F Zaina, S Negrini

**Affiliations:** 1 ISICO, Milan, Italy

## Background and purpose

Compliance to bracing has been questioned, and temperature sensors advocated to check it. Since 2010 we started the everyday clinical use of a temperature sensor (Thermobrace): aim of this study is to present the results of the first patients.

## Materials and methods

Population: 68 scoliosis consecutive patients (79% females, age 14.2±2.4) who accepted to use Thermobrace and had finished at least one period of treatment on the 31st December 2010 [1,2].

Actual hours worn per day were measured; compliance (percentage of prescription) and reported compliance (percentage of hours reported by the patient) were calculated. For reliability purposes, we use two different data processing methods.

## Results

Brace prescription was 16 to 23 hours per day. Average Thermobrace use was 5.25±2.25 months. Referred compliance was 94.3% (range 50-113%), the real one 86.1% (range 55-108%) or 89.9% (range 57-111%) according to the two different measurement methods. More than half of the patients had at least a 90% compliance with both readings. No wearing days were 1.0% of total and involved only 29% of patients.

## Conclusions

Compliance is neither due to type of treatment, nor to the patient alone. SOSORT criteria for bracing clearly state the importance of the treating team in this respect. This is the first study using a temperature sensor in a setting respecting SOSORT criteria, and shows compliance to bracing much higher than what was previously reported. In the everyday clinics, Thermobrace offers a valuable insight to increase compliance even further, and make treatment rely on real data.
